# Impact of nurse-delivered community-based CD4 services on facilitating pre-ART care in rural South Africa

**DOI:** 10.1186/s12913-016-1627-8

**Published:** 2016-08-11

**Authors:** T. Kompala, A. P. Moll, N. Mtungwa, R. P. Brooks, G. H. Friedland, S. V. Shenoi

**Affiliations:** 1 Department of Medicine, University of California, San Francisco, CA USA; 2 Church of Scotland Hospital, Tugela Ferry, South Africa; 3 Philanjalo NGO, Tugela Ferry, South Africa; 4 Yale University School of Medicine, Section of Infectious Diseases, AIDS Program, 135 College Street, Suite 323, New Haven, CT 06510 USA

**Keywords:** HIV/AIDS, HIV testing CD4 count staging, Community-based VCT, Nursing services, HIV decentralization, Task shifting

## Abstract

**Background:**

HIV testing, diagnosis and treatment programs have expanded globally, particularly in resource-limited settings. Diagnosis must be followed by determination of treatment eligibility and referral to care prior to initiation of antiretroviral treatment (ART). However, barriers and delays along these early steps in the treatment cascade may impede successful ART initiation. New strategies are needed to facilitate the treatment cascade. We evaluated the role of on site CD4+ T cell count phlebotomy services by nurses in facilitating pre-ART care in a community-based voluntary counseling and testing program (CBVCT) in rural South Africa.

**Methods:**

We retrospectively evaluated CBVCT services during five continuous time periods over three years: three periods when a nurse was present on site, and two periods when the nurse was absent. When a nurse was present, CD4 count phlebotomy was performed immediately after HIV testing to determine ART eligibility. When a nurse was absent, patients were referred to their local primary care clinic for CD4 testing. For each period, we determined the proportion of HIV-positive community members who completed CD4 testing, received notification of CD4 count results, as well as the time to test completion and result notification.

**Results:**

Between 2010 and 2013, 7213 individuals accessed CBVCT services; of these, 620 (8.6 %) individuals were HIV-positive, 205 (33.1 %) were eligible for ART according to South African national CD4 count criteria, and 78 (38.0 % of those eligible) initiated ART. During the periods when a professional nurse was available to provide CD4 phlebotomy services, HIV-positive clients were significantly more likely to complete CD4 testing than during periods when these services were not available (85.5 % vs. 37.3 %, *p* < 0.001). Additionally, when nurses were present, individuals were significantly more likely to be notified of CD4 results (60.6 % vs. 26.7 %, p <0.001). The time from HIV screening to CD4 test completion was also significantly shorter during nurse presence than nurse absence (median 8 days (IQR 4–19) vs. 35 days (IQR 15–131), *p* < 0.001).

**Conclusions:**

These findings indicate that in addition to CBVCT, availability of on site CD4 phlebotomy may reduce loss along the pre-ART care cascade and facilitate timely entry into HIV care.

## Background

The last decade has brought substantial expansion of HIV diagnosis and antiretroviral therapy (ART) programs to the developing world, yet major challenges persist. Rapid point-of-care HIV testing has allowed for decentralized diagnosis, even in resource-constrained settings. However, more than 50 % of those newly diagnosed with HIV are lost to follow up before initiating ART [1–4].

**Fig. 1:**
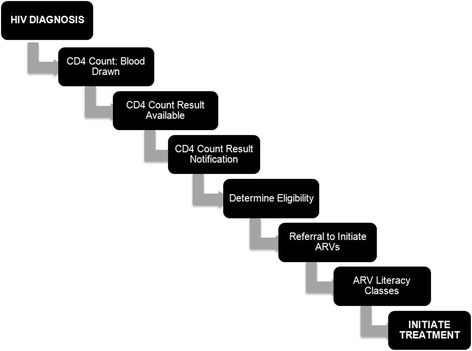
Pre-ART Care Cascade. After testing HIV-positive, multiple steps are required prior to initiating antiretroviral treatment. HIV-positive individuals are lost to care at each step of the cascade

HIV diagnosis is a critically important first step in the cascade of care, but many additional steps are required to successfully initiate ART (Fig. 1). Loss to pre-ART care occurs at each step. In the majority of sub-Saharan Africa, without a diagnosis of tuberculosis (TB) or World Health Organization (WHO) stage IV opportunistic infection, only HIV-positive individuals with a CD4+ T cell lymphocyte count below a certain threshold are eligible to initiate ART, [5] therefore CD4 cell count determination is a required step towards treatment initiation and therapeutic success [6]. After testing HIV-positive, individuals must provide venipuncture samples for CD4 testing, which are processed at centralized laboratories with flow cytometry capability. Results are communicated back to health providers, who must then locate patients to share the results and determine ART eligibility. Each of these steps requires multiple patient-initiated visits to the health care system, and must be successfully completed before an individual can begin treatment (Fig. 1). The time elapsed from HIV diagnosis to CD4 testing for eligibility determination can take weeks, even months, or patients may be lost to follow up altogether, with delays in ART initiation impacting morbidity and mortality [4, 6, 7].

Community-based intensive case finding with voluntary counseling and testing (CBVCT) for HIV has emerged as a strategy to expand access to HIV testing and improve linkage to HIV care, particularly in hard to reach populations. Similar to intensive case finding (ICF) for TB, active case finding for HIV may improve early diagnosis for those infected individuals who are not yet ill enough to present to hospitals and clinics, and may result in earlier ART initiation. Preliminary results from ongoing studies of CBVCT for HIV demonstrate promise of high diagnostic yield from these screening programs [8, 9].

Task shifting is a strategy to improve access to services in resource-limited settings plagued by health care worker shortages, [10–15] and is particularly relevant in South Africa, which is restructuring the health care system to emphasize primary care services [16, 17]. In response to the enormous scale of the HIV epidemic, South Africa has been progressive in training senior-level nurses (“Professional Nurses”) to initiate and monitor patients on ART, permitting expansion of HIV care particularly to rural or periurban primary care clinics [18]. Senior level nurses, however, are a scarce resource, especially in rural settings [19]. South African regulation has traditionally restricted phlebotomy to senior nurses, therefore requiring their continued involvement in CD4 testing for HIV staging.

We sought to determine the impact of community-based CD4 testing services within a CBVCT program that refers identified HIV-positive individuals into treatment programs in rural South Africa.

## Methods

### Study design & setting

We performed a retrospective review of de-identified programmatic data including 620 HIV-positive individuals identified within a community-based integrated HIV and TB intensive case finding program, operating from March 2010 to June 2013 in Tugela Ferry, Msinga sub-district of KwaZulu Natal province of South Africa. Msinga, a rural area with rugged terrain of nearly 2000 km^2^, has 180,000 traditional Zulu people, characterized by an extremely high antenatal HIV rate (>30 %), a heavy TB case notification rate burden (>1000/100,000) and high levels of poverty. The area is served by one district-level government hospital, 15 satellite primary health care clinics and 3 mobile clinics.

Since 2010, a local nongovernmental organization, in conjunction with the local Department of Health, has conducted integrated HIV and TB community-based VCT and intensive case finding. Teams of health educators, HIV counselors and a professional nurse travel to various congregate settings within Msinga to conduct health education and offer screening for HIV and TB. Screening locations include pension pay points (welfare grant delivery sites), municipality events, and taxi ranks, among others.

Adults (≥18 years old) who present to any CBVCT site are provided health education on HIV and TB in a group setting. Community members are offered and voluntarily come forward for rapid HIV fingerstick testing by a trained HIV counselor, with follow up on-site confirmatory rapid testing if positive. A professional nurse offers phlebotomy at the community site on the same day as HIV testing. Samples are submitted to the district hospital laboratory for flow cytometry analysis and CD4 results are generally available within 48 h. Once available, program staff notify community members. If a phone number was provided, telephone contact is attempted three times before pursuing notification in person. Physical notification is attempted twice and requires tracing by a trained staff member traveling in a vehicle with high road clearance to remote areas to locate patients’ homes. Whether by phone or in person, CD4 results are disclosed only to the patient, in a confidential setting, and counseling is provided. Patients are referred to local primary health clinics to initiate ART according to South African National Guidelines [5]; at the time of this program, HIV-infected individuals were ART eligible if CD4 count was less than 350 cells/mm^3^.

From March 2010 to June 2013, the CBVCT program operated continuously for HIV screening, but there were 3 periods in which professional nurses were consistently available at the screening sites, and 2 unanticipated periods in which they were not. This resulted in the opportunity to compare 5 sequential periods of program performance, with alternating periods of professional nurse presence and absence (Table 1). When a professional nurse was present on the CBVCT team, phlebotomy was performed on site on the day of HIV testing. When a nurse was absent from the CBVCT team, individuals found to be HIV-positive did not receive same day on-site phlebotomy for CD4 testing, and were referred to their local primary care clinics for CD4 testing with follow up by program staff. Nurse presence and nurse absence, therefore, determined the availability of same-day on-site phlebotomy services for CD4 testing.

**Table 1:** Baseline Characteristics of Five Different Periods of Nurse Presence or Absence

Baseline Character-istics	Period 1	Period 2	Period 3	Period 4	Period 5	Total	p-value
Professional Nurse	Present	Absent	Present	Absent	Present	--	--
Time Period	3/1/10–9/30/11	10/1/11–2/28/12	3/1/12–8/3/12	9/1/12–2/3/13	2/4/13–6/30/13		
Number of days	578	150	155	155	146	--	--
Number of People Screened	4200	728	1110	490	685	7213	--
Number (%) of HIV-positive	428 (10.2)	41 (5.6)	71 (6.4)	34 (6.9)	46 (6.7)	620 (8.6)	0.21
Median Age (IQR)	38 (28–48)	32 (27–46)	38.5 (27–48)	34 (29–47)	35.5 (27–41)	38 (28–48)	0.42
Females (%)	300 (70.1)	30 (73.2)	61 (85.9)	28 (82.4)	37 (80.4)	456 (73.5)	0.45
Number (%) receiving welfare grant	121 of 149^a^ (81.2)	35 (85.4)	64 (90.1)	30 (88.2)	45 (97.8)	295 of 341 (86.5)	0.33
Median CD4 count (IQR)	383 (252–550)	308 (245–541)	458 (288–551)	605 (473–813)	516 (381–694)	394 (260–564)	0.002
Number with phone (%)	348 (81.3)	34 (82.9)	59 (83.1)	25 (73.5)	42 (91.3)	508 (81.9)	0.14

^a^questions regarding welfare grants were added after the start of the program

### Data collection and analysis

This study retrospectively analyzed de-identified programmatic data from March 2010 to June 2013 as part of the ongoing community-based screening program. Demographics, HIV testing and treatment history were collected in a standardized screening questionnaire. CD4 test completion data, defined as completion of phlebotomy *and* laboratory reporting of CD4 result, was collected from clinics and laboratories in the study sub-district. The date of successful patient notification of CD4 results was also recorded. Result notification was defined as successful telephone contact, within three attempts, or in-person contact, within two attempts, to relay the results. Information on ART initiation was collected as part of the program monitoring and evaluation. Linkage to care and data on ART initiation was gathered from patient self-report or from clinics and depended on successful contact.

The primary outcomes were defined as proportion of HIV+ individuals who successfully completed CD4 testing, proportion of CD4 completers who were notified of CD4 count results, time to CD4 count completion from HIV testing, and time to result notification from HIV testing. These outcomes were compared between the five individual periods*,* and compared in aggregate between periods of nurse presence and periods of nurse absence. The Chi Square test was used to compare CD4 count completion and result notification between the two groups. T tests were used to compare mean time from screening to CD4 test completion, and mean time to result notification. Nonparametric testing was used to compare median times.

## Results

A total of 7213 individuals accessed screening services between March 2010 and June 2013; of these, 620 (8.6 %) individuals were HIV-positive (Table 1). Overall, the median age among HIV-positive community members was 38 years (IQR 28–48), 73.5 % were women, 85.6 % received a welfare grant from the government, and the median CD4 cell count was 394 (IQR 260–564). Characteristics of HIV-positive patients in the five defined sequential periods are summarized in Table 1. Period I was the longest and therefore also had the largest number of HIV-positive participants identified; this period also represents the longest continuous presence of a professional nurse on the CBVCT team.

**Fig. 2:**
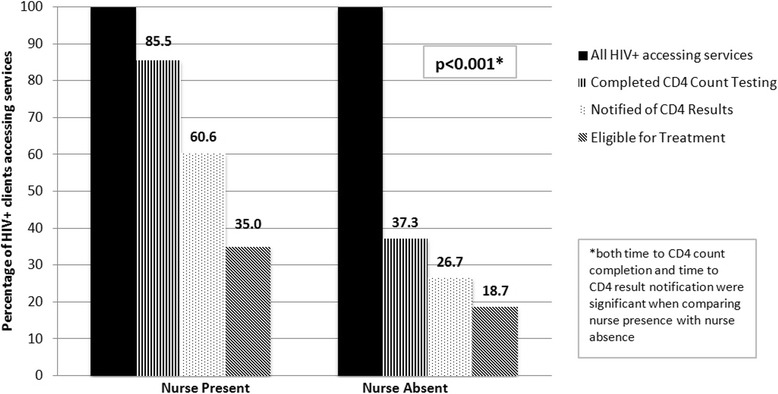
Successful Completion of Pre-ART Care During Nurse Presence vs. Nurse Absence. Comparing CD4 count completion and notification in aggregate across periods of nursing availability, HIV-positive individuals were significantly more likely to complete CD4 count staging when a nurse was present to provide community-based CD4 services

**Fig. 3:**
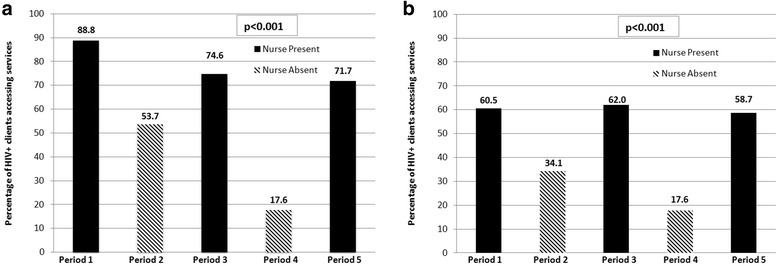
**a** and **b**. Successful CD4 Completion by Nurse Presence vs. Nurse Absence. Comparing individual periods of nursing availability, HIV-positive individuals were significantly more likely (**a**) to complete CD4 testing and (**b**) to receive CD4 count results when a nurse was part of the community-based VCT team

When a professional nurse was present as part of the CBVCT team and provided community-based CD4 services, HIV-positive clients accessing services were significantly more likely to complete CD4 testing than during periods when community-based CD4 services were not available (Fig. 2, 85.5 % vs. 37.3 %, *p* < 0.001). This difference remained significant for both aggregate findings (Fig. 2) during nurse presence and absence categories, and when comparing individual periods (Fig. 3a, except for Period 2 vs. Period 5 *p* = 0.08). Further, HIV-positive patients were successfully notified of their CD4 results significantly more frequently during nurse presence than during nurse absence (Fig. 3b, 60.6 % vs. 26.7 %, p <0.001). Result notification was significantly different when comparing the periods across both aggregate and individual periods.

**Fig. 4:**
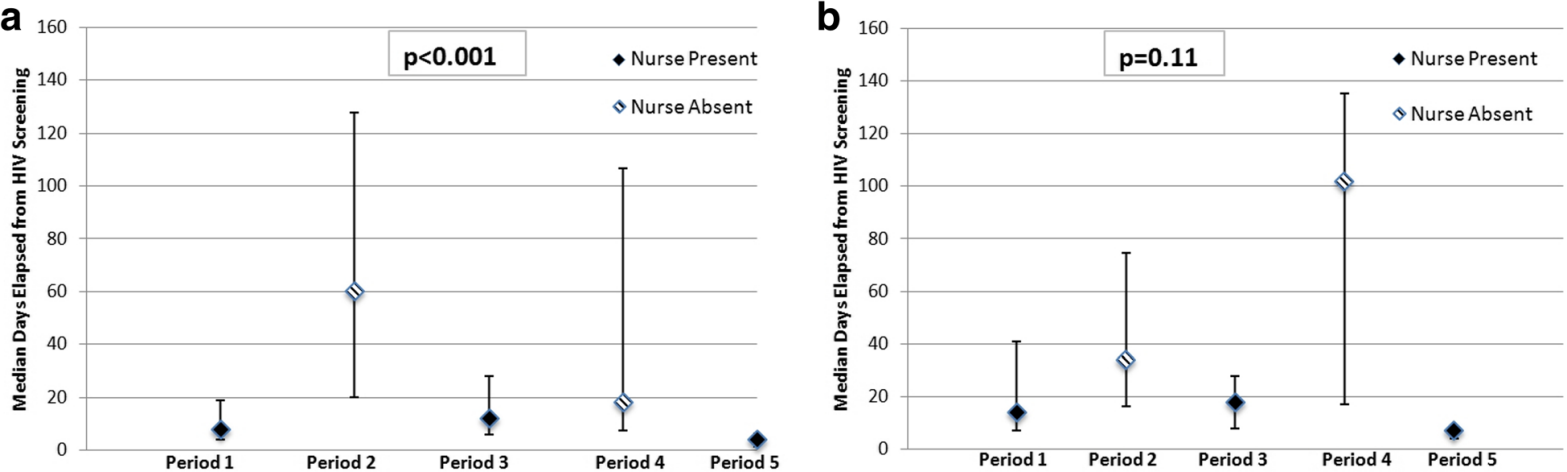
**a** and **b**. Time to CD4 Completion and Result Notification by Nurse Presence vs. Nurse Absence. Time to CD4 count completion was significantly shorter (**a**) when a nurse was providing services. Time to CD4 result notification was also shorter (**b**) when a nurse was present, but did not reach significance

Time from HIV screening to CD4 test completion was also significantly shorter during nurse presence than nurse absence (mean days 24.4 vs 72.4, median days 8 (IQR4-19) vs 35 (IQR15-131), *p* < 0.001, Fig. 4a). Similarly, time from HIV screening to successful CD4 result notification was shorter during nurse presence than nurse absence but did not reach statistical significance (mean days 47.7 vs. 81.6, median days 14 (IQR7-34) vs 47 (IQR16-119), *p* = 0.11, Fig. 4b).

In total, among 620 community members found to be HIV+ through our CBVCT service, 205 (33.1 %) were eligible for ART according to CD4 count criteria, and 78 (12.6 % of total and 38.0 % of those eligible) individuals initiated ART at their local clinic. Though there was no significant difference between number of days elapsed from HIV testing to ART initiation between periods of nurse presence and nurse absence (165 days vs. 100 days, *p* = 0.15), the overall time to ART initiation across all periods was quite lengthy (median 118 days, IQR 47–254).

## Discussion

We evaluated a CBVCT program in rural South Africa to determine the impact of providing on-site community-based CD4 count services at the site of HIV testing. In our model, professional nurses offered phlebotomy services for CD4 on the same day as HIV screening at community sites. Comparing periods of nurse absence and nurse presence at these sites was a surrogate for comparing same-day on site community-based CD4 testing against referral to local public health clinics for CD4 testing. We found that uptake and completion of CD4 testing was significantly higher when offered as part of community-based services as compared to clinic-based services.

Previous reviews [1, 2, 4] have examined pre-treatment loss to care in the HIV-positive population and demonstrated substantial loss of patients at every step between HIV diagnosis and ART initiation. This study reports the retention and loss-to-program of HIV-positive individuals at two steps (CD4 test completion and CD4 count result notification). Prior studies have considered HIV populations who seek care in clinic settings, but unique to this study is the patient population encountered entirely through a community-based VCT program. This study examines one CBVCT program over five alternating periods of nurse presence and unanticipated but continuous nurse absence, thus allowing us to determine the importance of nurse-provided community-based CD4 services while the remaining program characteristics were constant over time.

### CD4 count completion and result notification

Our results demonstrate that when community-based CD4 phlebotomy services are available at the time of HIV testing, significantly more HIV-positive individuals have CD4 testing *performed and completed* successfully. When nurses are absent and CD4 services are not available on the day of screening, individuals often do not complete CD4 testing and therefore are lost along the pre-ART care cascade.

Similarly, when a nurse was present and community-based CD4 services offered, the proportion of individuals who were *notified* of their results was also greater. When CD4 services are offered in the community setting, significantly more HIV-positive individuals successfully completed steps to move along the care cascade. Considering these two primary outcomes – CD4 test completion and CD4 result notification – this evaluation indicates that providing on site community-based CD4 services is critical for facilitating entry into HIV care.

### Time

In addition to completion of steps, we also address the time elapsed to step completion. Even after HIV diagnosis, substantial delays in eligibility determination and ART initiation may worsen morbidity and mortality, and also allow the possibility for continued HIV transmission [7]. This study shows that when nurses provided same-day community-based CD4 services, *the time to both CD4 count completion and time to result notification* were shorter, though the difference in the latter step was not statistically significant, likely due to small sample size. These data further support the role of community-based services to reduce loss along the cascade. This study also demonstrates that the time to ART initiation is quite protracted; regardless of the mechanism of completing CD4 staging, initiation of ART requires motivation by the patient to take action. Determining barriers to ART initiation after CD4 staging was beyond the scope of this study but is essential to expediting ART initiation.

### Role of nurses

With respect to our CBVCT program, professional nurses were essential for completing on-site phlebotomy for CD4 count. In the absence of a professional nurse, individuals identified as HIV-positive required referral to their local clinic for CD4 cell phlebotomy. Our results demonstrate that these community members frequently did not follow up, representing a lost opportunity for facilitating entry into care for HIV. Although HIV screening and diagnosis continue even when a nurse is not available, our ability to help HIV-positive individuals move beyond the first step is limited in such cases. ART eligibility determination and subsequent initiation hinge on successful CD4 testing completion and result notification, both of which were markedly improved with community-based CD4 services.

The alternating periods of nurse absence and presence occurred naturally in an unplanned manner, and are reflective of the difficulty in recruiting and retaining professional nurses in rural areas and for community-level work. The present health care worker (HCW) shortage in South Africa is perhaps further exaggerated when HCWs are less inclined to work in rural areas in potentially more arduous conditions in the community in favor of working in urban areas or in health care facilities.

### Challenges in patient communication

In the present system, blood samples are collected in the community but sent for CD4 analysis in a distant lab. Health care providers are therefore required to continue communication with the patient in the days after screening to successfully notify them of results. Though 80 % of all HIV-positive individuals encountered in this CBVCT program were able to provide a phone number, successful post-screening communication was difficult. For those who could not be reached by phone after three attempts, and for those who did not provide a phone number, staff members were required to travel into the community to find patients and notify them of results. The realities of poverty, limited telephone access, poor network reception, and lack of electricity, coupled with rugged terrain made patient notification a challenging and resource intensive process – further impeding successful contact between health care provider and patient in the critical days after determining an HIV-positive diagnosis.

### A role for point-of-Care CD4 analysis

This study has demonstrated the impact of providing on site community-based CD4 phlebotomy services, yet we have also demonstrated the challenges of sustaining a professional nurse to offer these services. How can mobile CBVCT programs improve their ability to provide community-based CD4 services? Point-of-care (POC) CD4 technology may provide an answer. During this project, only traditional flow cytometry was used for CD4 analysis to determine ART eligibility. In recent years, however, low-cost POC testing options for CD4 count have emerged to address a critical need for rapid diagnostics in resource-limited settings [20]. Use of POC CD4 testing is growing, especially in clinic settings [21], but its use in mobile community-based screening services in rugged, resource-limited terrain has been limited. Reliable accuracy and appropriate quality control measures need to be further evaluated in this setting.

As this study has demonstrated, providing community-based CD4 services significantly decreases loss along the cascade, compared against referral to clinic for CD4 testing. POC testing could potentially further improve successful progress along the cascade by facilitating CD4 testing, result notification, and determination of ART eligibility status all on the same day and at the same location as HIV testing. When laboratory based CD4 testing is used, the patient leaves CBVCT services with a diagnosis but with uncertainty about severity of disease or eligibility for starting treatment. Having immediate knowledge of CD4 count results after HIV diagnosis would permit patient-centered counseling, may improve patients’ ability to understand disease severity, and facilitate rapid entry into HIV care to initiate ART. Further studies should evaluate the effect of immediate receipt of CD4 counts after community-based HIV diagnosis on effective transition to ART initiation.

This study demonstrated improved CD4 notification rates when a nurse was present, suggesting that nurses are important in notifying and counseling patients on these results. Similar to their role in conducting HIV testing and counseling and supporting treatment adherence, non-clinical personnel may be able to contribute to CD4 result notification and may be able to be trained to perform fingerstick POC CD4 testing in order to preserve scarce senior level nurses for higher level tasks and patient care in line with WHO-endorsed task shifting efforts [15, 22–25].

### Limitations

This evaluation was a retrospective observational study and was limited to using only routinely collected program information. The periods of nurse presence and nurse absence were not controlled, and were dependent on successful recruitment and retention of professional nurses for the CBVCT program. While we assume remaining program characteristics did not change, other confounders may have affected our results. Data collection itself was not biased by presence or absence of nurse; screening information was collected and entered by trained field health workers and therefore was independent of professional nurse involvement. CD4 test completion data derives only from clinics and laboratories in the study subdistrict. If a patient completed CD4 testing outside of the geographic region where they reside, that information could not be captured. Information on ART initiation was from patient self-report or from their designated nearest clinic; It is possible that individuals with whom we have not made successful contact, completed CD4 testing and initiated ART at another clinic. Rates of ART initiation in this study group may be underestimated. ART initiation was not a primary outcome of our study but was part of an internal monitoring and evaluation assessment; due to small sample size, we were unable to make conclusions about community-based CD4 testing on ART initiation. Future studies should evaluate barriers to ART initiation facing patients after CD4 count staging. Larger and prospective studies are needed to follow HIV+ patients in resource-limited settings through the entire treatment cascade to more precisely document the impact of each step in the cascade toward ART initiation and the desired therapeutic outcome – sustained viral suppression.

## Conclusion

Providing community-based CD4 services significantly improves CD4 completion and notification, reduces loss along the pre-ART care cascade, and enables decentralized HIV care. In impoverished, rural areas where patient notification and access to care are particularly challenging and nursing human resources are scarce, offering POC CD4 assays within community-based services may facilitate improved pre-ART care.

## Abbreviations

ART, antiretroviral treatment; CBVCT, community based voluntary counseling and testing; HCW, healthcare worker; ICF, intensive case finding; POC, point of care; TB, tuberculosis; WHO, World Health Organization.

## References

[1] Rosen S, Fox MP (2011). Retention in HIV care between testing and treatment in sub-Saharan Africa: a systematic review. PLoS Med.

[2] Mugglin C, Estill J, Wandeler G, Bender N, Egger M, Gsponer T (2012). Loss to programme between HIV diagnosis and initiation of antiretroviral therapy in sub-Saharan Africa: systematic review and meta-analysis. Tropical Med Int Health.

[3] Losina EBI, Giddy J, Chetty S, Regan S, Walensky RP, Ross D, Scott CA, Uhler LM, Katz JN, Holst H, Freedberg KA (2010). The "ART" of linkage: pre-treatment loss to care after HIV diagnosis at two PEPFAR sites in Durban, South Africa. Plos One.

[4] Kranzer KGD, Ford N, Johnston V, Lawn SD (2012). Quantifying and addressing losses along the continuum of care for people living with HIV infection in sub-Saharan Africa: a systematic review. J Int AIDS Soc.

[5] Republic of South Africa Department of Health. South African Antiretroviral Treatment Guidelines. South African Department of Health; 2010.

[6] Larson BA, Brennan A, McNamara L, Long L, Rosen S, Sanne I (2010). Early loss to follow up after enrolment in pre-ART care at a large public clinic in Johannesburg, South Africa. Tropical Med Int Health.

[7] Bassett IV, Wang B, Chetty S, Mazibuko M, Bearnot B, Giddy J (2009). Loss to care and death before antiretroviral therapy in Durban, South Africa. J Acquir Immune Defic Syndr.

[8] Khumalo Sakutukwa G, Morin S, Fritz K, Charlebois E, van Rooyen H, Chingono A (2008). Project Accept (HPTN 043): a community-based intervention to reduce HIV incidence in populations at risk for HIV in sub-Saharan Africa and Thailand. J Acquir Immune Defic Syndr.

[9] Sweat M, Morin S, Celentano D, Mulawa M, Singh B, Mbwambo J (2011). Community-based intervention to increase HIV testing and case detection in people aged 16–32 years in Tanzania, Zimbabwe, and Thailand (NIMH Project Accept, HPTN 043): a randomised study. Lancet Infect Dis.

[10] Emdin CA, Chong NJ, Millson PE (2013). Non-physician clinician provided HIV treatment results in equivalent outcomes as physician-provided care: a meta-analysis. J Int AIDS Soc.

[11] Crisp N, Chen L (2014). Global supply of health professionals. N Engl J Med.

[12] Zachariah R, Ford N, Philips M, Lynch S, Massaquoi M, Janssens V (2009). Task shifting in HIV/AIDS: opportunities, challenges and proposed actions for sub-Saharan Africa. Trans R Soc Trop Med Hyg.

[13] Alliance GHW, editor Community Health Workers and other Front Line Health Workers: Moving from Fragmentation to Syngergy to achieve Universal health Coverage. Third Global Forum on Human Resources for Health; 2013

[14] Cometto GWS (2013). Tackling health workforce challenges to universal health coverage: setting targets and measuring progress. Bull World Health Organ.

[15] World Health Organization (2008). Task shifting: rational redistribution of tasks among health workforce teams: global recommendations and guidelines.

[16] Fairall L, Bachmann MO, Lombard C, Timmerman V, Uebel K, Zwarenstein M (2012). Task shifting of antiretroviral treatment from doctors to primary-care nurses in South Africa (STRETCH): a pragmatic, parallel, cluster-randomised trial. Lancet.

[17] Republic of South Africa Department of Health. Human Resources for Health Strategy for the Health Sector, South Africa: 2012/2013-2016/2017. South African Department of Health, 2011.

[18] Nyasulu JC, Muchiri E, Mazwi SL, Ratshefola M (2013). NIMART rollout to primary healthcare facilities increases access to antiretrovirals in Johannesburg: an interrupted time series analysis. S Afr Med J.

[19] George G, Gow J, Bachoo S (2013). Understanding the factors influencing health-worker employment decisions in South Africa. Hum Resour Health.

[20] Rowley CF (2014). Developments in CD4 and Viral Load Monitoring in Resource-Limited Settings. Clin Infect Dis.

[21] Jani I, Sitoe N, Chongo P, Alfai E, Quevedo J, Tobaiwa O (2011). Accurate CD4 T-cell enumeration and antiretroviral drug toxicity monitoring in primary healthcare clinics using point-of-care testing. AIDS.

[22] Uwimana J, Zarowsky C, Hausler H, Swanevelder S, Tabana H, Jackson D (2013). Community-based intervention to enhance provision of integrated TB-HIV and PMTCT services in South Africa. Int J Tuberc Lung Dis.

[23] Lewin S, Munabi-Babigumira S, Glenton C, Daniels K, Bosch-Capblanch X, van Wyk BE, et al. Lay health workers in primary and community health care for maternal and child health and the management of infectious diseases. The Cochrane Database of Systematic reviews. 2010; (3):Cd004015. 10.1002/14651858.CD004015.pub3 PMC6485809 20238326

[24] Selke HM, Kimaiyo S, Sidle JE, Vedanthan R, Tierney WM, Shen C (2010). Task-shifting of antiretroviral delivery from health care workers to persons living with HIV/AIDS: clinical outcomes of a community-based program in Kenya. J Acquir Immune Defic Syndr.

[25] Dudley L, Garner P. Strategies for integrating primary health services in low- and middle-income countries at the point of delivery. Cochrane Database Syst Rev. 2011;(7):Cd003318. 10.1002/14651858.CD003318.pub3 PMC6703668 21735392

